# Deep Learning-Based Adaptive Neural-Fuzzy Structure Scheme for Bearing Fault Pattern Recognition and Crack Size Identification

**DOI:** 10.3390/s21062102

**Published:** 2021-03-17

**Authors:** Farzin Piltan, Bach Phi Duong, Jong-Myon Kim

**Affiliations:** Department of Electrical, Electronics and Computer Engineering, University of Ulsan, Ulsan 44610, Korea; piltanfarzin@gmail.com (F.P.); duongbachphi@gmail.com (B.P.D.)

**Keywords:** autoregressive-Laguerre method, support vector regression technique, high-order variable structure observer, adaptive neural-fuzzy technique, convolution neural network, support vector machine, bearing, fault pattern recognition, crack size identification

## Abstract

Bearings are complex components with onlinear behavior that are used to mitigate the effects of inertia. These components are used in various systems, including motors. Data analysis and condition monitoring of the systems are important methods for bearing fault diagnosis. Therefore, a deep learning-based adaptive neural-fuzzy structure technique via a support vector autoregressive-Laguerre model is presented in this study. The proposed scheme has three main steps. First, the support vector autoregressive-Laguerre is introduced to approximate the vibration signal under normal conditions and extract the state-space equation. After signal modeling, an adaptive neural-fuzzy structure observer is designed using a combination of high-order variable structure techniques, the support vector autoregressive-Laguerre model, and adaptive neural-fuzzy inference mechanism for normal and abnormal signal estimation. The adaptive neural-fuzzy structure observer is the main part of this work because, based on the difference between signal estimation accuracy, it can be used to identify faults in the bearings. Next, the residual signals are generated, and the signal conditions are detected and identified using a convolution neural network (CNN) algorithm. The effectiveness of the proposed deep learning-based adaptive neural-fuzzy structure technique by support vector autoregressive-Laguerre model was analyzed using the Case Western Reverse University (CWRU) bearing vibration dataset. The proposed scheme is compared to five state-of-the-art techniques. The proposed algorithm improved the average pattern recognition and crack size identification accuracy by 1.99%, 3.84%, 15.75%, 5.87%, 30.14%, and 35.29% compared to the combination of the high-order variable structure technique with the support vector autoregressive-Laguerre model and CNN, the combination of the variable structure technique with the support vector autoregressive-Laguerre model and CNN, the combination of RAW signal and CNN, the combination of the adaptive neural-fuzzy structure technique with the support vector autoregressive-Laguerre model and support vector machine (SVM), the combination of the high-order variable structure technique with the support vector autoregressive-Laguerre model and SVM, and the combination of the variable structure technique with the support vector autoregressive-Laguerre model and SVM, respectively.

## 1. Introduction

Rotary machines are used in many industries for numerous purposes. Bearings are one of the main components used in rotating machines to reduce friction, and they are used as components in robots, turbines, and various types of motors. Identifying the operating conditions of these components can be of particular importance for industries, and early anomaly detection can play a decisive role in diagnosing the condition of a bearing [1]. Bearings are exposed to four types of defects: inner, outer, roller, and cage. Condition monitoring is the first step for bearing anomaly detection and includes multiple procedures, such as vibration, acoustic emission, and motor current signature analysis [2,3].

Fault diagnosis occurs as part of condition monitoring, which is a subset of control engineering. There are three fundamental procedures for anomaly detection in bearings. The first method comprises data-driven techniques that use only the data collected by sensors and analyzes these data using signal processing and machine/deep learning techniques [4,5,6,7]. The second approach is model-based, in that the system is modeled using various algorithms, and the fault can be detected and classified using the difference between data extracted from the sensors and the system’s model [8,9]. Third, hybrid algorithms combine data-driven and model-based approaches for anomaly detection and classification in systems [10,11,12].

Model-based and data-driven techniques each have their own positive and negative aspects regarding bearing anomaly detection. Data-driven techniques work based on the analysis of data collected by sensors, and reliability is one of their main drawbacks. On the other hand, model-based approaches are generally reliable; complexity is the foremost issue of the model-based approach [2,3]. Thus, a hybrid strategy is recommended in this research paper. In this work, model-based, deep learning, and artificial intelligence schemes are combined for fault pattern recognition and crack size identification in bearings.

To detect anomalies using the model-based technique, modeling is the critical step. System modeling is categorized into two principal groups: (a) mathematical-based system modeling, such as Newton–Euler, Lagrange, and finite element methods, and (b) system identification approaches, such as autoregressive (AR), autoregressive with external input (ARX), ARX–Laguerre techniques, neural network approach, and fuzzy logic methods [13,14,15,16,17,18,19,20,21,22,23,24,25]. To improve the accuracy of signal approximation in nonlinear and nonstationary signals, we propose a combination of autoregressive techniques, namely the Laguerre technique with support vector regression (SVR), which will henceforth be called the support vector autoregressive–Laguerre (SVAL).

After approximating the vibration signal of the bearing in the normal state, an estimator is designed to solve the signal estimation problems in the state-space modeling technique. Estimators can be categorized into linear (such as proportional integral (PI) [16,19] and proportional multi-integral [26]) and nonlinear (such as high gain [27], feedback linearization [28], neural network [29], fuzzy logic [30,31], and sliding mode [32,33,34,35,36]) observers. In this work the high order variable structure observer is recommended for pattern recognition and crack size identification. The adaptive neural-fuzzy inference technique is suggested to reduce the estimation error of the signal and have discriminative signals in various conditions. Therefore, the combination of the HOVSO with an adaptive neural-fuzzy inference system (ANFIS), which will henceforth be called an adaptive neural-fuzzy structure observer (ANFSO), is recommended for signal estimation.

As recently as ten years ago, conventional machine learning-based classifiers for fault analysis, such as K-nearest neighbor [37], support vector machine (SVM) [38], and multilayer perceptron [39], had emerged as the prevalent and powerful techniques to solve the issues of fault diagnosis for the rotary machine’s health monitoring since they have the competence to gain valuable fault information from large datasets. However, one of the main limitations of these approaches is that these classifiers with shallow structure rely on the precise feature engineering that solves the selectivity-invariance dilemma, wherein the features must be expertly designed. This is often a difficult task, especially when considering the nonlinear system characteristics. Another issue of the conventional machine learning classifiers is they are not able to be improved with increasing the training data size. Recently, deep learning (DL) methods have been receiving more attention as a new approach for fault diagnosis because they require less need for feature engineering and can obtain high performance with a large amount of data. The DL algorithm consists of multiple blocks of layer units with the non-linear operation and demonstrates a capability to provide an automatic method for deep extracting and suitable selecting useful features which contain rich knowledge of the fault from the signal. Deep learning algorithms, such as stacked auto-encoders [40], and the convolutional neural network (CNN) [41], adversarial reconstruction CNN [42], and vision-based three dimensional structure [43], have been investigated in fault detection studies. Unlike the stacked auto-encoder, the CNN utilizes an architecture in form of end-to-end learning model that does not need the pre-training stage for each layer. With the higher performance of CNN-based classifiers for the diagnosis accuracy, selecting an optimal CNN model in bearing fault diagnosis is indispensable to obtain the faulty information from the vibration signals, and then increase consistency, and diagnosis accuracy. To adapt with the estimated residual signal from the observation technique in form of the 1-D matrix, we propose using the 1-D CNN with a specific structure as the decision-making method to perform the classification with high fault diagnosis accuracy, in diagnosing multiple faults under the different severity level of bearing. Hence, the degradation level of bearing with different crack sizes of each type of fault is able to be predicted in our proposed method.

This study makes the following contributions:

- The combination of autoregressive technique, Laguerre method, and support vector regression is used for vibration signal approximation of a bearing.
- The combination of higher order variable structure technique, support vector autoregressive–Laguerre, and adaptive neural-fuzzy inference technique is suggested for signal estimation under different conditions.
- The normal data are used for training the modeling and estimation and the proposed algorithm is applied to an unknown dataset.
- The support vector autoregressive–Laguerre and adaptive neural-fuzzy structure observer is combined with CNN for fault pattern recognition and crack size identification in one frame.

The structure of this article is as follows. Related work is described in the second section. The Case Western Reserve University (CWRU) bearing dataset is described in the second section. In the third section, the proposed method for vibration signal approximation, signal estimation, and classification is introduced. The results are discussed in Section 4. Finally, the conclusions are provided in Section 5.

## 2. Related Work

Several techniques have been introduced as hybrid schemes, including the combination of machine/deep learning and signal processing, the combination of model-based techniques and signal processing, and the combination of model-based techniques and machine/deep learning [10,11,12].

The combination of signal processing and deep learning was introduced in [44]. In this research, in the first stage, the hybrid feature pool is generated using an envelope spectrum, time domain, and wavelet packet transform, next, the stacked autoencoder is used to perform fault diagnosis. The main drawback of this research is the number of features and selecting the best features.

The combination of a data-driven technique and control algorithm for fault diagnosis were presented in [16]. In this work, the system was modeled by the ARX–Laguerre technique and the PI observer was recommended for signal estimation. This technique was recommended for a 2nd order system but in vibration non-stationary signals this technique has challenges.

To improve the performance of the above challenge, the hybrid technique based on a model-based method integrated with a deep learning algorithm was proposed in [45]. The rotor signal was modeled and estimated in the first stage using an autoregressive–Laguerre proportional integral observer. Next, the estimated signal was prepared by resampling and frequency transform. Then, the scalable deep neural network was used for the fault decision. The challenge of vibration signal modeling can be addressed by the mathematical-based system modeling five degrees of freedom vibration bearing modeling. Mathematical-based system modeling (such as five degrees of freedom vibration bearing modeling) is reliable but has some drawbacks, such as the lack of complexity and uncertainty related to modeling [13,14,15]. Linear-based system identification techniques (such as the combination of autoregressive with external inputs, and autoregressive with external inputs and Laguerre technique) have been used to address the above challenges [15,16,17,18,19]. Artificial intelligence methods (such as various kinds of neural networks and fuzzy logic procedures) have been used for nonlinear-based system modeling. For example, applications of fuzzy logic and neural network techniques in system modeling are mentioned in [20,21,22,23,24,25], respectively.

To increase the accuracy of the PI observer, the proportional multi-integral observer was presented in [26]. In this work, the PI observer was used to estimate the original signals, and the next integral term was suggested to reduce the effect of uncertainties. To improve the robustness of the PI observer, the PI observer integrated with the sliding mode technique was presented in [19]. In linear estimators, the gain updating factors (such as the proportional and integral coefficients) are used to fine-tune the signal estimation, whereas in nonlinear estimators, in addition to the gain updating factors, nonlinear behavior extracted from the nonlinear system model is used to fine-tune the signal estimation and reduce the estimation error [27]. One nonlinear signal estimator is the feedback linearization technique. The most consequential obstacle of this technique is the severe dependency on the system’s dynamic model and robustness, especially in highly uncertain conditions [28]. The candidate for solving the problems of the feedback linearization algorithm is the variable structure estimator [32,33,34,35]. This technique provides a lower estimation error due to its higher robustness. Unfortunately, the variable structure technique has the challenge of high-frequency oscillation (chattering phenomenon), which increases the error of estimation. To address this issue, a low-pass filter was used to extract fault information [36]. Nevertheless, if the filter’s parameters are not selected appropriately, it may have problems in fault identification and crack size detection. Consequently, the accuracy of signal estimation for anomaly detection and identification decreases. To reduce the chattering and increase the estimation accuracy, the combination of high-order and super-twisting with the variable structure algorithm was recommended in [35].

## 3. Proposed Scheme

**Figure 1:**
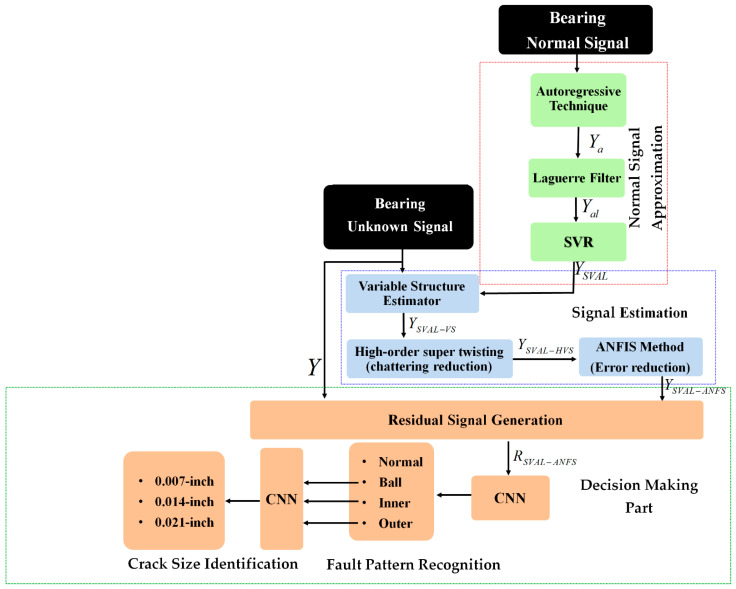
Bearing fault pattern recognition and crack size identification using the proposed combination of support vector autoregressive-Laguerre, adaptive neural-fuzzy structure technique, and convolution neural network (CNN). SVR: support vector regression; ANFIS: adaptive neural-fuzzy inference system.

Therefore, in this study, the combination of the SVAL and the ANFSO with a 1D-CNN is recommended for fault pattern recognition and crack-size identification. To achieve these purposes, there are three stages: (a) normal signal approximation, (b) signal estimation, and (c) fault pattern recognition and crack size identification, as shown in Figure 1.

In the first stage, as shown in Figure 1, the SVAL combination is recommended for the approximation of the normal vibration signal. Therefore, first, the autoregressive technique is used to model the vibration signals under normal conditions. Next, the Laguerre technique is used to strengthen the autoregressive technique. To increase the accuracy of the autoregressive-Laguerre scheme, the support vector regression is introduced.

In the second part, as shown in Figure 1, ANFSO is recommended for signal estimation and to prepare discriminative signals under various conditions. So, first, the variable structure estimator is suggested. To overcome the nonlinear part of the vibration signal, the support vector autoregressive-Laguerre from the modeling part is borrowed for this part and strengthens the signal estimation property. Next, the combination of the variable structure estimator with a high-order super-twisting technique is used to reduce the high-frequency oscillation in the variable structure technique. Next, to increase the fault pattern recognition and crack size identification accuracy, the combination of variable structure estimator and high-order super-twisting technique with ANFIS is suggested. Finally, as shown in Figure 1, the 1D-CNN is recommended to classify the residual signals. In this step, first, the residual signal is calculated as the difference between the original and estimated signals using the proposed estimation algorithm. Next, the pattern of residual signals is recognized, and the crack sizes are identified by a 1D-CNN.

### 3.1. Test Bench and Data Collection

**Figure 2:**
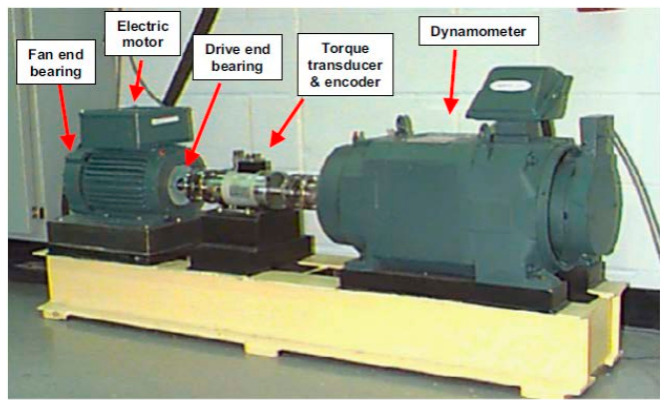
Case Western Reverse University (CWRU) data acquisition center [35,46].

The CWRU dataset is selected to test the proposed adaptive hybrid observation-based algorithm. In this dataset, a two-horsepower (hp) induction motor is utilized to rotate the bearing at various speeds [46]. A vibration sensor collects normal and abnormal vibration signals with a sampling rate of 48 kHz. The bearing used in the CWRU dataset is the 6205-2RS JEM SKF roller bearing. Moreover, four different states are defined in this dataset: normal condition (NRM), ball fault (BLF), inner race fault (IRF), and outer race fault (ORF). Additionally, the abnormal conditions (e.g., ball fault, inner race fault, and outer race fault) have three different crack sizes: 0.007 in, 0.014 in, and 0.021 in. The vibration signal in the normal condition when the torque load is 0 hp is modeled and estimated. Next, all conditions of signals including normal and abnormal (e.g., ball fault, inner fault, and outer fault) in various torque loads (e.g., 0 hp, 1 hp, 2 hp, and 3 hp) and different crack sizes (0.007 in, 0.014 in, and 0.021 in) are used for test, fault pattern recognition, and crack size identification. Figure 2 illustrates the CWRU test bench for data acquisition. Table 1 summarizes the data contained in the CWRU dataset [46].

### 3.2. Support Vector Autoregressive–Laguerre Signal Approximation

The development of the ANFSO for fault pattern recognition and crack size identification is at the heart of this work. In the design of this observer, the signal approximation technique is the main part. Therefore, the SVAL technique is introduced for signal approximation. First, the autoregressive algorithm is used to approximate the normal RAW signal of the bearing [16,19,47].

(1) $$\{\begin{array}{c} X_{a}\left(k+1\right)\;=\;\left[\alpha_{a}X_{a}\left(k\right)\;+\;U_{a}\left(k\right)\right]\;+\;e_{a}\left(k\right)\\ Y_{a}\left(k\right)\;=\;{\left(\alpha_{o}\right)}^{T}X_{a}\left(k\right) \end{array}.$$

Here, $X_{a}\left(k\right)$ is the state of the bearing vibration signal using the autoregressive technique, $U_{a}\left(k\right)$ is the uncertainty of the bearing vibration signal based on the autoregressive point of view, $e_{a}\left(k\right)$ is the error of the bearing vibration signal modeling using the autoregressive technique, $Y_{a}\left(k\right)\;$is the output measurable state of the bearing vibration signal using the autoregressive technique, and $\left(\alpha_{a},\alpha_{o}\right)$ are the coefficients for state and output. The uncertainty and the error of signal modeling based on the autoregressive technique for the vibration signal are defined using the following equations, respectively:

(2) $$\{\begin{array}{c} U_{a}\left(k\right)\;=\;Y\left(k\right)\;-\;Y_{a}\left(k\right)\\ e_{a}\left(k\right)\;=\;Y_{a}\left(k\;+\;1\right)\;-\;Y_{a}\left(k\right) \end{array}.$$

Here, Y(k) is the original normal RAW signal. To improve the robustness and increase the performance, the autoregressive technique is combined with the Laguerre algorithm [16,19]. The combination of Laguerre and autoregressive techniques (henceforth called AL), the uncertainty approximation using the AL method, and the error of the AL technique are represented by the following state-space algorithms, respectively [16,19,47].

(3) $$\{\begin{array}{c} X_{al}\left(k\;+\;1\right)\;=\;\left[\alpha_{a}X_{al}\left(k\right)\;+\;U_{al}\left(k\right)\;+\;\alpha_{alo}Y_{al}\left(k\right)\right]\;+\;e_{al}\left(k\right)\\ Y_{al}\left(k\right)\;=\;{\left(\alpha_{o}\right)}^{T}X_{al}\left(k\right) \end{array},$$

(4) $$\{\begin{array}{c} U_{al}\left(k\right)\;=\;Y\left(k\right)\;-\;Y_{al}\left(k\right)\\ e_{al}\left(k\right)\;=\;Y_{al}\left(k\;+\;1\right)\;-\;Y_{al}\left(k\right) \end{array}.$$

Here, $X_{al}\left(k\right)$ is the state of the bearing vibration signal using the AL technique, $e_{al}\left(k\right)$ is the error of the bearing vibration signal approximation using the AL technique, $U_{al}\left(k\right)$ is the uncertainty of the bearing vibration signal approximation based on the AL technique, $Y_{al}\left(k\right)$ is the output state of the bearing vibration signal using the AL technique, and $\left(\alpha_{alo}\right)$ is the coefficient of the combination of the Laguerre and autoregressive techniques for output. To increase the performance of signal approximation and to cover the nonlinear behavior of the Lyapunov-based observer, SVAL is recommended.

The SVR is a machine learning technique used to approximate the vibration bearing signal. This technique is defined by the following equation [48].

(5) $$Y_{SVR}\;=\;\underset{i}{\sum }\left(\alpha_{i}{}^{+}\;-\;\alpha_{i}{}^{-}\right)K\left(x_{i},x\right)\;+\;b$$

Here, $Y_{SVR}$ is the output modelled flowrate based on SVR, $\left(\alpha_{i}{}^{+},\alpha_{i}{}^{-}\right)$ are the Lagrange coefficients, $K\left(x_{i},x\right)$ is the kernel, and b is the bias. Various functions can be introduced as kernel functions; in this work, the Gaussian function is selected and defined as follows.

(6) $$K\left(x_{i},x\right)\;=\;e^{\left(-\frac{1}{2\sigma^{2}}{\left\|x_{i}-x\right\|}^{2}\right)}$$

Here, σ is variance. So, we have:

(7) $$min\underset{i}{\sum }\underset{j}{\sum }\left(\alpha_{i}{}^{+}\;-\;\alpha_{i}{}^{-}\right)\left(\alpha_{i}{}^{+}\;-\;\alpha_{i}{}^{-}\right)K\left(x_{i},x\right)$$

Then $K\left(x_{i},x\right)$ is defined by $w_{ij}\;$and

(8) $$min\underset{i}{\sum }\underset{j}{\sum }\alpha_{i}{}^{+}\alpha_{i}{}^{+}w_{ij}\;-\;\alpha_{i}{}^{-}\alpha_{i}{}^{+}w_{ij}\;-\;\alpha_{i}{}^{+}\alpha_{i}{}^{-}w_{ij}\;+\;\alpha_{i}{}^{-}\alpha_{i}{}^{-}w_{ij}$$

While $W\;=\;\left[w_{ij}\right]\in {\mathbb{R}}^{n\times n},\;\alpha \;=\;{\left[\begin{array}{c} \alpha^{+}\\ \alpha^{-} \end{array}\right]}_{2n\times 1},\;\varpi \;=\;\left[\begin{array}{cc} W & -W\\ -W & W \end{array}\right]$. Therefore, the above formulation is rewritten as

(9) $$min\frac{1}{2}\alpha^{T}\varpi \alpha \;+\;\kappa^{T}\alpha$$

$\kappa ={\left[\begin{array}{c} -Y\;+\;\varepsilon \\ Y\;+\;\varepsilon \end{array}\right]}_{2n\times 1}$. Here, Y is the vibration signal and ε is accepted boundary of modeling.

(10) $$min\frac{1}{2}\alpha^{T}\varpi \alpha \;+\;\kappa^{T}\alpha$$

Moreover, the bias is represented using the following equation.

(11) $$b\;=\;\frac{1}{\left|S\right|}\underset{s\in S}{\sum }\left[Y_{s}\;-\;\underset{i\in S}{\sum }\left(\alpha_{i}{}^{+}\;-\;\alpha_{i}{}^{-}\right)\;\times \;K\left(x_{i},x_{S}\right)\;-\;\varepsilon \;\times \;sign\left(\alpha_{i}{}^{+}\;-\;\alpha_{i}{}^{-}\right)\right]$$

Here, $Y_{s}$ is the signal of support vector and S is support vector. The support vector is represented by the following equation.

(12) $$S\;=\;\left\{i|0\;<\;\alpha_{i}{}^{+}\;+\;\alpha_{i}{}^{-}\;<\;\delta \right\}$$

Here, δ is a constant. The SVAL algorithm is represented by the following definitions.

(13) $$\{\begin{array}{c} X_{SVAL}\left(k\;+\;1\right)\;=\;\left[\alpha_{a}X_{SVAL}\left(k\right)\;+\;U_{SVAL}\left(k\right)\;+\;\alpha_{lao}\left(Y_{al}\left(k\right)\;+\;Y_{SVR}\left(k\right)\right)\right]\;+\;e_{SVAL}\\ Y_{SVAL}\left(k\right)\;=\;{\left(\alpha_{o}\right)}^{T}X_{SVAL}\left(k\right) \end{array},$$

(14) $$\{\begin{array}{c} U_{SVAL}\left(k\right)\;=\;Y\left(k\right)\;-\;Y_{SVAL}\left(k\right)\\ e_{SVAL}\left(k\right)\;=\;Y_{SVAL}\left(k\;+\;1\right)\;-\;Y_{SVAL}\left(k\right) \end{array}.$$

Here, $X_{SVAL}\left(k\right)$ is the state of bearing vibration signal using the SVAL algorithm; $e_{SVAL}\left(k\right)\;$is the error of bearing vibration signal modeling using the SVAL algorithm; $U_{SVAL}\left(k\right)$ is the uncertainty approximation using the SVAL algorithm; $Y_{SVAL}\left(k\right)\;$is the output measurable state of the bearing vibration signal using the SVAL algorithm, and $\left(Y_{SVR}\right)$ is the uncertainty state of the bearing vibration signal approximation using support vector regression technique, respectively.

### 3.3. Deep Learning-Based Adaptive Neural-Fuzzy Structure Observer for Fault Pattern Recognition and Crack Size Identification

Regarding Figure 1, first, the bearing vibration signal in the normal state was modeled using the SVAL, and the state-space equation of the vibration signal under normal conditions was extracted using Equation (13). In this section, first, an adaptive hybrid observer is recommended for normal and abnormal signals estimation; second, the residual signal, which is the difference between RAW and estimated bearing signals, is generated, and finally, the CNN is represented for fault pattern recognition and crack size identification in the bearing.

For signal estimation, first, the variable structure observer is recommended. After this, to reduce the fluctuation and chattering phenomenon, the higher order technique is suggested. The adaptive neural-fuzzy inference technique is used to improve the estimation accuracy in the normal condition and have discriminative signals in various conditions. Therefore, the ANFSO is recommended for signal estimation.

This part has two main sub-sections: (a) ANFSO based on the combination of HOVSO and ANFIS to estimate the bearing signals, and (b) residual signal generation and fault decision using, first, generation of the residual signal and second, fault pattern recognition and crack size identification using the CNN.

#### 3.3.1. Adaptive Neural-Fuzzy Structure Observer

Based on Figure 1, in this part, ANFSO is presented for signal estimation. The variable structure technique is a robust observer for signal estimation. Based on Equations (13) and (14), and [14], the state-space equation for variable structure observer is defined using the following equations.

(15) $$\{\begin{array}{c} X_{SVAL-VS}\left(k\;+\;1\right)\;=\;[\alpha_{a}X_{SVAL-VS}\left(k\right)\;+\;U_{SVAL-VS}\left(k\right)\;+\;\alpha_{lao}(Y_{SVAL-VS}\left(k\right)+\\ +\;Y_{SVR}\left(k\right))]\;+\;e_{SVAL}\left(k\right)\;+\;\alpha_{VS}sgnU_{SVAL-VS}\\ Y_{SVAL-VS}\left(k\right)\;=\;{\left(\alpha_{o}\right)}^{T}X_{SVAL-VS}\left(k\right) \end{array}$$

(16) $$\{\begin{array}{c} U_{SVAL-VS}\left(k\right)\;=\;\alpha_{p}\left(Y\left(k\right)\;-\;Y_{SVAL-VS}\left(k\right)\right)\;+\;\alpha_{VS}sgn(Y\left(k\right)\;-\;Y_{SVAL-VS}\left(k\right))\\ e_{SVAL}\left(k\right)\;=\;Y_{SVAL}\left(k\;+\;1\right)\;-\;Y_{SVAL}\left(k\right) \end{array}.$$

Here, $X_{SVAL-VS}\left(k\right)$ is the state of bearing vibration signal using the combination of the SVAL algorithm for approximation and variable structure technique for estimation; $e_{SVAL}\left(k\right)\;$is the error of bearing vibration signal modeling using the SVAL algorithm; $U_{SVAL-VS}\left(k\right)$ is the uncertainty approximation using the combination of the SVAL algorithm for approximation, and the variable structure technique for estimation; $Y_{SVAL-VS}\left(k\right)$ is the output measurable state of the bearing vibration signal using the combination of the SVAL algorithm for approximation and the variable structure technique for estimation, and $\left(\alpha_{VS},\alpha_{p}\right)$ is the coefficient of the variable structure technique. To reduce the effect of the chattering phenomenon, the high-order variable structure observer is recommended.

(17) $$\psi \;=\;\alpha_{1}{\left\|Y\left(k\right)\;-\;Y_{SVAL-VS}\left(k\right)\right\|}^{0.5}\;+\;\alpha_{2}sgn(Y\left(k\right)\;-\;Y_{SVAL-VS}\left(k\right))$$

Here, ψ is the new high-order variable structure observation and $\left(\alpha_{1},\alpha_{2}\right)$ are coefficients. The super-twisting definition is defined by the following equation.

(18) $$\{\begin{array}{c} \alpha_{1}{\left\|Y\left(k\right)\;-\;Y_{SVAL-VS}\left(k\right)\right\|}^{0.5}\;+\;\alpha_{2}\;\times \;sgn(Y\left(k\right)\;-\;Y_{SVAL-VS}\left(k\right))\;-\;\rho \\ \dot{\rho }\;=\;\alpha_{3}\;\times \;sgn(Y\left(k\right)\;-\;Y_{SVAL-VS}\left(k\right)) \end{array}.$$

Here, $\dot{\rho }$ is the super-twisting variable and $\left(\alpha_{3}\right)$ is the respective coefficient. In an uncertain condition, this technique is used to reduce the estimation error and moves towards zero in a finite time. Therefore, the combination of the SVAL algorithm for approximation and the high-order super variable structure technique for estimation of the vibration bearing signal are presented as the following equations.

(19) $$\{\begin{array}{c} X_{SVAL-HVS}\left(k\;+\;1\right)\;=\;[\alpha_{a}X_{SVAL-HVS}\left(k\right)\;+\;U_{SVAL-HVS}\left(k\right)\;+\\ \alpha_{lao}\left(Y_{SVAL-HVS}\left(k\right)\;+\;Y_{SVR}\left(k\right)\right)]\;+\;e_{SVAL}\left(k\right)\;+\;\alpha_{VS}\;\times \;sgn{\left|U_{SVAL-HVS}\right|}^{0.75}\\ Y_{SVAL-HVS}\left(k\right)\;=\;{\left(\alpha_{o}\right)}^{T}X_{SVAL-HVS}\left(k\right) \end{array}$$

(20) $$\{\begin{array}{c} U_{SVAL-HVS}\left(k\right)\;=\;\alpha_{p}(Y(k)\;-\;Y_{SVAL-HVS}\left(k\right))\;+\;\alpha_{VS}sgn(Y(k)\;-\\ Y_{SVAL-HVS}(k))\;+\;\alpha_{1}{\left\|Y\left(k\right)\;-\;Y_{SVAL-HVS}(k)\right\|}^{0.5}\;+\;\alpha_{2}\times sgn(Y(k)\;-\\ Y_{SVAL-HVS}(k))\;-\;\;\rho \\ \dot{\rho }\;=\;\alpha_{3}\;\times \;sgn(Y(k)\;-\;Y_{SVAL-HVS}\left(k)\right)\\ e_{SVAL}(k)\;=\;Y_{SVAL}(k\;+\;1)\;-\;Y_{SVAL}(k) \end{array}$$

Here, $X_{SVAL-HVS}\left(k\right)$ is the state of bearing vibration signal using the combination of the SVAL algorithm for approximation and high-order super twisting variable structure technique for estimation; $e_{SVAL}\left(k\right)$ is the error of bearing vibration signal modeling using the SVAL algorithm; $U_{SVAL-HVS}\left(k\right)$ is the uncertainty approximation using the combination the SVAL algorithm for approximation and high-order super twisting variable structure technique for estimation, and $Y_{SVAL-HVS}\left(k\right)$ is the output measurable state of the bearing vibration signal using the combination of the SVAL algorithm for approximation and the high-order super twisting variable structure technique for estimation. To improve the flexibility and accuracy of signal estimation in the presence of uncertainties, the ANFSO is used. The ANFIS procedure is recommended to reduce the effect of uncertainties [49]. Moreover, the uncertainty performance index (UPI) for the combination of the SVAL algorithm for approximation and high-order super twisting variable structure technique for estimation, Equation (20), is represented by the following function.

(21) $${UPI}_{SVAL-HSV}\;=\;\frac{1}{k}\overset{k}{\underset{1}{\sum }}{\left(Y_{SVAL-HVS}\;-\;Y\right)}^{2}$$

Here, ${UPI}_{SVAL-HSV}\;$is the UPI using the combination of the SVAL algorithm for approximation and high-order super twisting variable structure technique for estimation. To minimize the ${UPI}_{SVAL-HSV}$, the ANFIS technique is recommended in this work. First, the Takagi-Sugeno-Kang (TSK) fuzzy logic method is represented using the following definition.

(22) $$U_{ANFIS}\left(k\right)\;=\;\frac{\sum_{r}U_{r}\;\times \;\gamma_{r}}{\sum_{r}\gamma_{r}},\;\;\gamma_{r}\;=\;\underset{r}{\sum }e^{(-0.5\sum_{i}\left(\frac{X\left(k\right)-\beta_{r}}{\delta }{)}^{2}\right)}.$$

Here, $U_{ANFIS}\left(k\right)$ is the uncertainty performance estimation using the ANFIS technique, $\beta_{r}$ is the membership function selection, and δ is variance. The UPI in the ANFIS technique can be represented by the following definition.

(23) $${UPI}_{ANFIS}\;=\;\frac{{\left(\sum_{1}^{k}\left(U_{r}\left(k\right)\;-\;U_{r}\right)\;\times \;\gamma_{r}\right)}^{2}}{{\left(\sum_{1}^{k}\gamma_{r}\right)}^{2}}$$

To minimize ${UPI}_{ANFIS}$ based on the gradient descent method, we have

(24) $$\begin{array}{c} ({UPI}_{ANFIS}){\;}_{min}\;=\;\frac{\partial }{\partial \beta_{r}}\;\times \;{\left(\overset{k}{\underset{1}{\sum }}\left(U_{r}\left(k\right)\;-\;U_{r}\right)\;\times \;\gamma_{r}\right)}^{2}\;+\;\frac{\partial }{\partial \gamma_{r}}\;\times \\ {(\overset{k}{\underset{1}{\sum }}\left(U_{r}\left(k\right)\;-\;U_{r}\right))}^{2}\frac{\partial \gamma_{r}}{\partial \beta_{r}}\;=\;2\left(U_{r}\left(k\right)\;-\;U_{r}\right)\;\times \;\gamma_{r}\left(U_{r}\left(k\right)\;-\;U_{r}\right)\;\times \;\gamma_{r}\;\times \;\frac{\partial \gamma_{r}}{\partial \beta_{r}} \end{array}$$

Therefore, by updating the $\beta_{r}\;$and δ, the ${UPI}_{ANFIS}\;$can be minimized. This means that the accuracy and performance of TSK fuzzy logic, which is defined in Equation (22), is improved. So, the adaptive $\beta_{r}\;$and δ are defined by the following equation.

(25) $$\beta_{r,t+1}\;=\;\beta_{r,t}\;-\;\Theta_{r,t}\frac{\partial UPI_{ANFIS}}{\partial \beta_{r,t}}$$

(26) $$\delta_{t+1}\;=\;\delta_{t}\;-\;\Theta_{r,t}\frac{\partial UPI_{ANFIS}}{\partial \delta_{t}}$$

Here, $\Theta_{r,t}\;$is the tuning coefficient. Therefore, regarding Figure 1, ANFSO is defined using the following definitions.

(27) $$\{\begin{array}{c} X_{SVAL-ANFS}\left(k\;+\;1\right)\;=\;[\alpha_{a}X_{SVAL-ANFS}\left(k\right)\;+\;U_{SVAL-ANFS}\left(k\right)+\\ \alpha_{lao}\left(Y_{SVAL-ANFS}\left(k\right)\;+\;Y_{SVR}\left(k\right)\right)]\;+\;e_{SVAL}\left(k\right)+\\ \alpha_{VS}\;\times \;sgn{\left|U_{SVAL-ANFS}\right|}^{0.75}\\ Y_{SVAL-ANFS}\left(k\right)\;=\;{\left(\alpha_{o}\right)}^{T}X_{SVAL-ANFS}\left(k\right) \end{array}.$$

(28) $$\{\begin{array}{c} U_{SVAL-ANFS}\left(k\right)\;=\;\alpha_{p}Y\left(k\right)\;-\;Y_{SVAL-ANFS}\left(k\right))\;+\;\alpha_{VS}sgn(Y\left(k\right)\\ -Y_{SVAL-ANFS}\left(k\right))\;+\;\alpha_{1}{\left\|Y\left(k\right)\;-\;Y_{SVAL-ANFS}(k)\right\|}^{0.5}\;+\\ \alpha_{2}\;\times \;sgn(Y(k)\;-\;Y_{SVAL-ANFS}(k))\;+\;\alpha_{ANFIS}U_{ANFS}(k)\;-\;\rho \\ \dot{\rho }\;=\;\alpha_{3}\;\times \;sgn(Y(k)\;-\;Y_{SVAL-HVS}\left(k)\right)\\ e_{SVAL}(k)\;=\;Y_{SVAL}(k\;+\;1)\;-\;Y_{SVAL}(k) \end{array}.$$

Here, $X_{SVAL-ANFS}\left(k\right)$ is the state of bearing vibration signal using the combination of the SVAL algorithm for approximation and ANFSO for estimation, $e_{SVAL}\left(k\right)$ is the error of bearing vibration signal modeling using the SVAL algorithm; $U_{SVAL-ANFS}\left(k\right)$ is the uncertainty approximation using the combination of the SVAL algorithm for approximation and ANFSO for estimation, $Y_{SVAL-ANFS}\left(k\right)$ is the output state of the bearing vibration signal using the combination of the SVAL algorithm for approximation and ANFSO for estimation, and $\alpha_{ANFIS}$ is the state coefficient.

In summary, this sub-section consisted of three main parts. First, the variable structure observer was designed. To reduce the chattering phenomenon, in the second stage, the variable structure observer was improved using the high-order super twisting variable structure observer. Finally, the combination of the high-order super twisting variable structure observer and adaptive neural-fuzzy inference system was introduced to improve the flexibility and accuracy of signal estimation. For the fault decision, the next sub-section focuses on generating the residual signal and implementing the CNN.

#### 3.3.2. Fault Pattern Recognition and Crack Size Identification

As shown in Figure 1, the normal signal was approximated using a combination of autoregressive technique, Laguerre approach, and support vector regression method. Next, the combination of high-order variable structure technique, support vector autoregressive–Laguerre, and adaptive neural-fuzzy inference algorithm was designed to improve the power of the signal estimation technique. In this section, the residual signal is specified. The residual signal is the difference between the original and estimated signals. Based on this definition, the residual signals for (a) the combination of the SVAL algorithm and variable structure technique, (b) the combination of the SVAL algorithm and high-order variable structure technique, and (c) the combination of the SVAL algorithm and adaptive neural-fuzzy structure method are represented by Equations (29)–(31), respectively.

(29) $$R_{SVAL-VS}\left(k\right)\;=\;Y\;-\;Y_{SVAL-VS}\left(k\right)$$

(30) $$R_{SVAL-HVS}\left(k\right)\;=\;Y\;-\;Y_{SVAL-HVS}\left(k\right)$$

(31) $$R_{SVAL-ANFS}\left(k\right)\;=\;Y\;-\;Y_{SVAL-ANFS}\left(k\right)$$

Here, $R_{SVAL-VS}\left(k\right)$ is the residual signal based on the combination of the SVAL algorithm and variable structure technique, $R_{SVAL-HVS}\left(k\right)$ is the residual signal based on the combination of the SVAL algorithm and high-order variable structure method, and $R_{SVAL-ANFS}\left(k\right)$ is the residual of the bearing signals based on the combination of the SVAL algorithm and adaptive neural-fuzzy structure method. Regarding these equations, $Y_{SVAL-VS}\left(k\right),Y_{SVAL-HVS}\left(k\right),$ and $Y_{SVAL-ANFS}\left(k\right)$ are calculated using Equation (15), (19), and (27), respectively.

After obtaining the residual signals, they will be fed to the 1D-CNN to classify the signals to the respective classes. A 1D-CNN operates with a given input signal $S_{j-1}^{\left(k\right)}$ with K channels from the previous layer j−1, a convolutional layer calculates an $m^{th}$ output of the feature map as the convolutional operation of the input and the network’s parameters which present the filters with the weight matrix and bias vector. So that the adjusted variables of the network are formulated as a sequence of filters. Different from the conventional artificial neural network with multi-layer perceptron, each neuron in a convolutional layer is only connected to a small group of neurons in the previous layer, which reduces the computational complexity of the CNN compared to the full-connected structure in terms of matrix multiplication operation. The weights and biases in the filter are shared and distributed over the local area of input matrix, which effectively captures and exploits the local spatial characteristics network layers and enables the CNN to lattice the layer data with less computation to facilitate feature extraction. The convolutional operation can be described as:

(32) $$Conv_{j}^{\left(m\right)}\;=\;f\left(\overset{K}{\underset{k=1}{\sum }}W_{j}^{\left(k,m\right)}*S_{j-1}^{\left(k\right)}\;+\;B_{j}^{\left(m\right)}\right)$$

**Figure 3:**
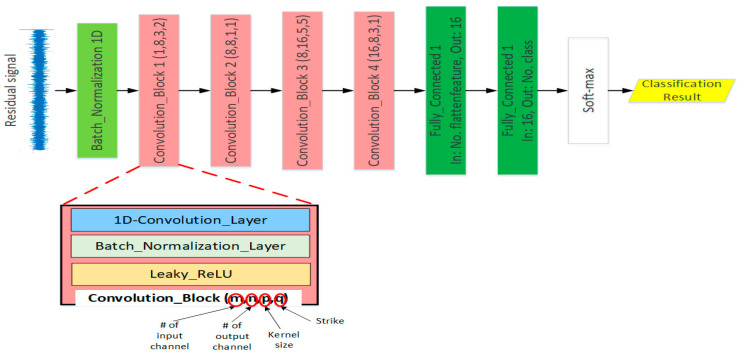
The structure of 1D-CNN for classification.

Here, j presents for the convolutional layer’s order in the network. The convolution operation of the input $S_{j-1}$ with the weight matrix $W_{j}^{\left(k,m\right)}$ and the bias vector $B_{j}^{\left(m\right)}$, which yield the $m^{th}$ output of the feature map, is represented by the (∗) operator. K stands for the number of channels in each signal. Then, the sum of convolutions plus a bias vector is put into the nonlinear activation function f to obtain the output of the current layer. The residual input signal is a vector that has a size of 1200 samples with one channel. After each convolution step, the feature of the bearing fault in the residual signal is automatically extracted in the form of high-level abstract information. After the convolutional layer, batch normalization is employed to elevate the convergence of the training process by rising the capacity of regularization for the model and it also avoids the overfitting phenomenon. Batch normalization can reduce the impacts of earlier layers by keeping the mean and variance fixed. Finally, the batch normalization sub-layer’s output is fed as the input to the leaky-ReLU nonlinear activation function. Figure 3 illustrates the designed architecture of the 1D-CNN model with the convolutional blocks and fully connected layers. The model comprises four convolution blocks with various numbers of filters to extract the information, i.e., 1–8, 8–8, 8–16, and 16–8 for each block, respectively. Each block illustrates one feature learning step that includes three sub-layers of convolution, batch-normalization, and activation function. After each convolution block, the features will become more abstract. After the output of the fourth convolutional blocks, the abstract feature maps are flattened and put into the two fully connected layers and one soft-max layer. The two fully connected layers and the soft-max layer play the role of classification of the residual signal into the respective classes for fault pattern recognition and crack size identification. Various optimization constraints, comprising Xavier initialization methods, batch normalization, dropout, and leaky-ReLU (leaky rectified linear unit), are also incorporated into the basic model of the 1D-CNN to perform better classification accuracy. The most regularly utilized functions of non-linear activation includes the hyperbolic tangent, the sigmoid and the ReLU (rectified linear unit). However, the leaky-ReLU function has been indicated to be more efficacious than the other activation functions because it allows the proposed model to obtain sparse representations in a simple way in comparison with the sigmoid and hyperbolic tangent, and it also solves the issue of a dying ReLU function when great values of gradient flow across it. Conventionally, the CNN structure applied the pooling layer to decrease the number of neural in the feature maps by using the subsampling operator to reduce the number of optimizable parameters. Thus, it quickens the computation time for the 1D-CNN. In this research, we considered using the convolution layers with a large size of kernel and big strike step, instead of using pooling to reduce the size of spatial feature maps.

For each type of condition, the residual signal is made up of 120,000 values. It was segmented into 100 samples; each sample contains 1200 values. The training set includes 80% of the data sample and the testing set includes the remaining 20%. The details of the training and testing dataset are presented in Table 2.

The networks are trained with a stochastic gradient descent for 25 epochs with a learning rate α = 0.001 and a batch size 10. The research employed the Adam (adapted moment estimation), which is established as a back-propagation strategy, to control the learning rate and other hyperparameters of the network structure during the training phase. The loss function that is minimized during the training process is the categorical cross-entropy loss function. Algorithm 1 presents the proposed algorithm, which is the combination of the SVAL algorithm and adaptive neural-fuzzy structure with CNN.
**Algorithm 1.** The proposed scheme: The combination of the SVAL algorithm and adaptive neural-fuzzy structure with CNN.1:**Signal Modeling** Approximate the bearing function from normal vibration signal using autoregressive technique. (1,2)2:Improve the robustness of autoregressive technique by combining autoregressive algorithm with the Laguerre filter. (3,4)3:Increase the accuracy and nonlinearity of Equation (3) using support vector autoregressive–Laguerre (SVAL). (13,14)4:**Signal Estimation** ASignal estimation using the combination of the SVAL algorithm and variable structure technique. (15,16)5:Reduce the chattering of variable structure technique using the combination of the SVAL algorithm and high-order variable structure technique. (19,20)6:Increase the stability and accuracy of high-order variable structure technique using the combination of the SVAL algorithm and adaptive neural-fuzzy structure (proposed method). (27,28)
**Fault Decision**7:Generate the residual signal. (31)8:Resample the residual signals. 9:Fault pattern recognition and crack size identification using 1D-CNN. (32)

## 4. Results

**Figure 4:**
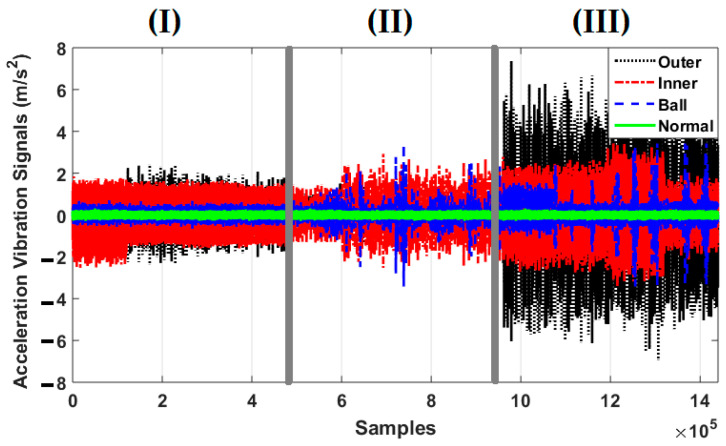
Bearing vibration signals for four fault conditions—normal, ball fault, inner fault, and outer fault—for crack sizes of (**I**) 0.007-inch, (**II**) 0.014-inch, and (**III**) 0.021-inch.

To test the power of fault pattern recognition and crack size identification, the CWRU dataset was used in this work. This vibration dataset has four classes: normal, ball fault, inner fault, and outer fault. Figure 4 illustrates the original vibration signals for the four classes. Regarding this figure, when the crack size is 0.007 in, inner and outer faults have overlapping vibration signals; when the crack size is 0.014 in, inner, outer, and ball faults have overlapping vibration signals; for 0.021 in crack size, inner and ball faults have overlapping vibration signals. These overlaps in vibration signals reduce the classification accuracy. The difference between the original RAW signal of a bearing in normal condition when the torque load and crack size are 0 hp and 0.007 in, respectively, based on the autoregressive technique, the combination of autoregressive and Laguerre technique, and the SVAL algorithm were defined based on Equations (2), (4) and (14), respectively.

**Figure 5:**
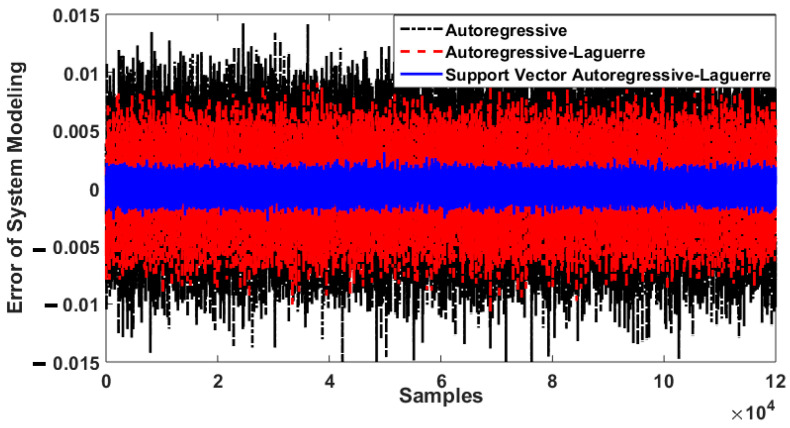
Bearing modeling error using autoregressive technique, autoregressive–Laguerre method, and the support vector autoregressive–Laguerre approach.

Figure 5 shows the bearing modeling error based on the autoregressive technique, the combination of autoregressive and Laguerre technique, and the SVAL algorithm. It is clear that the bearing modeling error in the SVAL approach is lower than in the other two techniques. In the next section, the combination of ANFSO and CNN is used for estimation of the vibration signals, fault pattern recognition, and crack size identification in the bearing. To test the power of fault pattern recognition and crack size identification using the combination of the SVAL algorithm and adaptive neural-fuzzy structure (proposed) + CNN (proposed + CNN), this procedure is validated and compared with the following state-of-the-art techniques: (a) the combination of the SVAL algorithm and variable structure technique + CNN (SVAL-VSO + CNN), (b) the combination of the SVAL algorithm and high-order variable structure technique + CNN (SVAL-HVSO + CNN), (c) the original RAW signal + CNN (RAW + CNN), (d) the combination of the SVAL algorithm and adaptive neural-fuzzy structure (proposed) + SVM (SVAL-ANFSO + SVM), (e) the combination of the SVAL algorithm and variable structure technique + SVM (SVAL-VSO + SVM), and (f) the combination of the SVAL algorithm and high-order variable structure technique + SVM (SVAL-HVSO + SVM).

**Figure 6:**
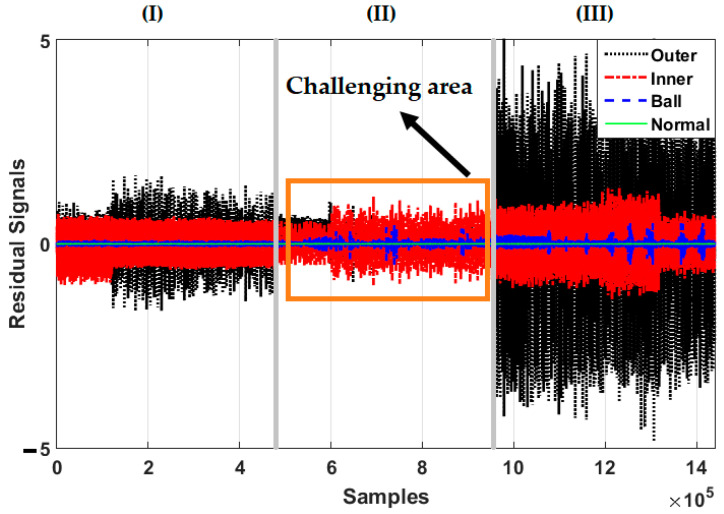
Residual signals for four conditions—normal, ball fault, inner fault, and outer fault—based on the combination of the SVAL algorithm and variable structure method for crack sizes of (**I**) 0.007-inch, (**II**) 0.014-inch, and (**III**) 0.021-inch.

**Figure 7:**
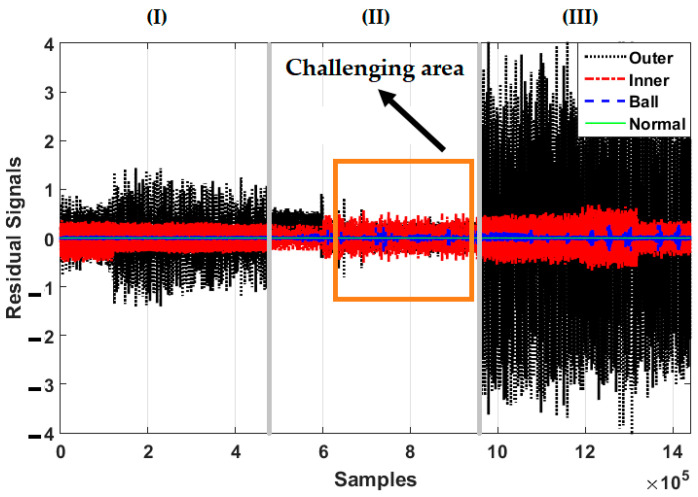
Residual signals for four conditions—normal, ball fault, inner fault, and outer fault—based on the combination of the SVAL algorithm and high-order variable structure method for crack sizes of (**I**) 0.007-inch, (**II**) 0.014-inch, and (**III**) 0.021-inch.

**Figure 8:**
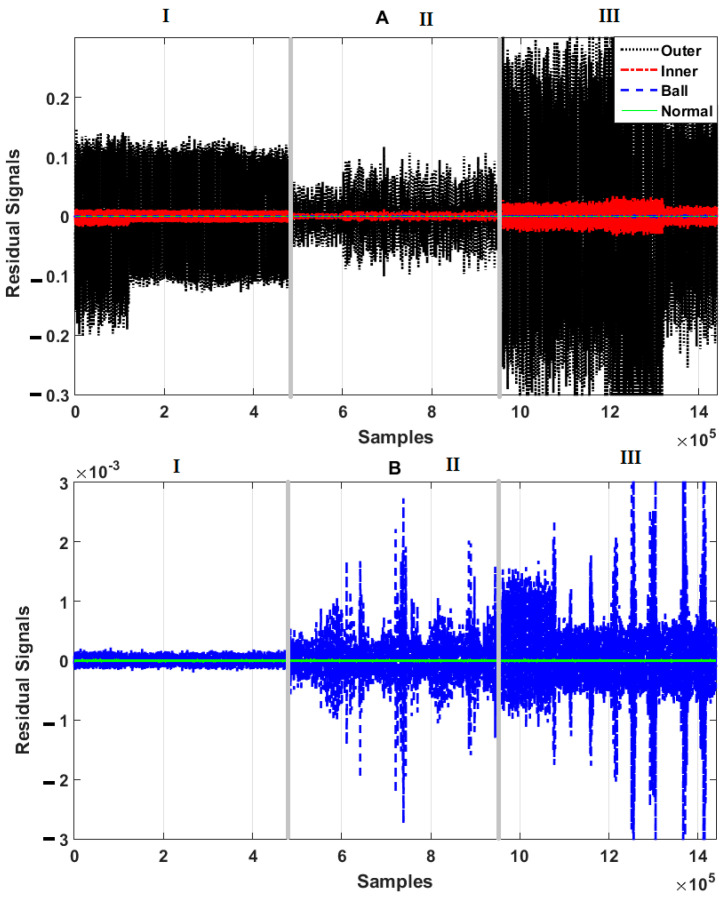
Residual signals for four conditions—normal, ball fault, inner fault, and outer fault—based on the combination of the SVAL algorithm and adaptive neural-fuzzy structure method for crack sizes of (I) 0.007-inch, (II) 0.014-inch, and (III) 0.021-inch. (**A**): normal view, (**B**): zoom view.

Figure 6, Figure 7 and Figure 8 illustrate the residual signals for the healthy, ball, inner, and outer conditions in three different crack sizes—0.007 in, 0.014 in, and 0.021 in—based on the combination of the SVAL algorithm and variable structure technique, the combination of the SVAL algorithm and high-order variable structure technique, and the combination of the SVAL algorithm and adaptive neural-fuzzy structure (i.e., the proposed) method, respectively.

Regarding Figure 6, when the crack size is 0.014 in, the overlap between inner and outer faults is increased. This problem reduces the classification accuracy based on the combination of the SVAL algorithm and variable structure technique. Based on this figure, it is observed that this technique faces a challenge in terms of crack size identification.

Figure 7 shows the power of the combination of the SVAL algorithm and high-order variable structure technique for fault pattern recognition and crack size identification. As highlighted in this figure, however, this technique improves on the performance of the combination of the SVAL algorithm and variable structure technique, but it has limitations related to fault pattern recognition and crack size identification. Figure 8 shows the power of the combination of the SVAL algorithm and adaptive neural-fuzzy structure (proposed) method for fault pattern recognition and crack size identification. As seen in this figure, the combination of the SVAL algorithm and adaptive neural-fuzzy structure method is able to distinguish different conditions and faults based on the residual signals for fault pattern recognition and crack size identification better than the other two methods.

**Figure 9:**
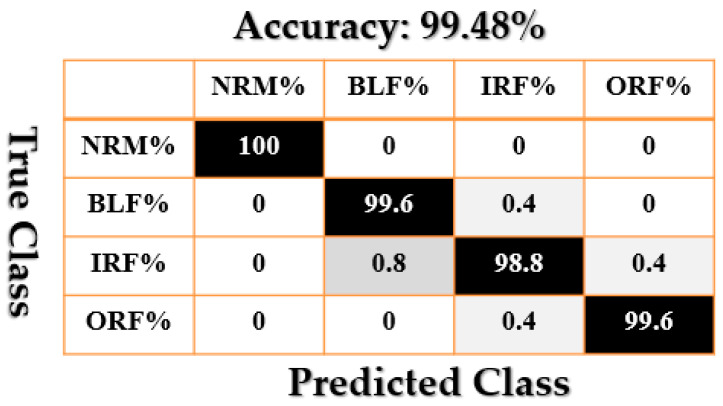
Fault pattern recognition confusion matrix based on the proposed + CNN.

**Figure 10:**
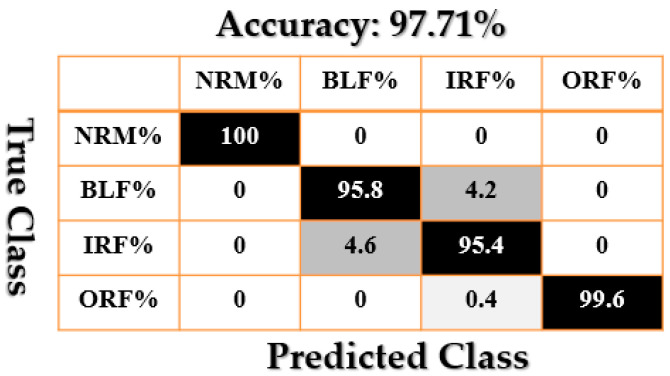
Fault pattern recognition confusion matrix based on the SVAL-HVSO + CNN.

**Figure 11:**
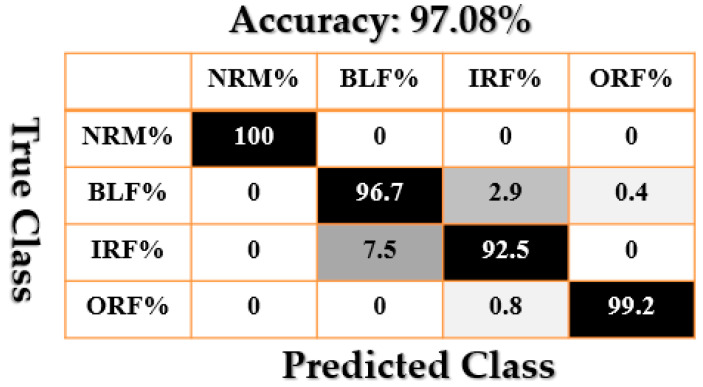
Fault pattern recognition confusion matrix based on SVAL-VSO + CNN.

**Figure 12:**
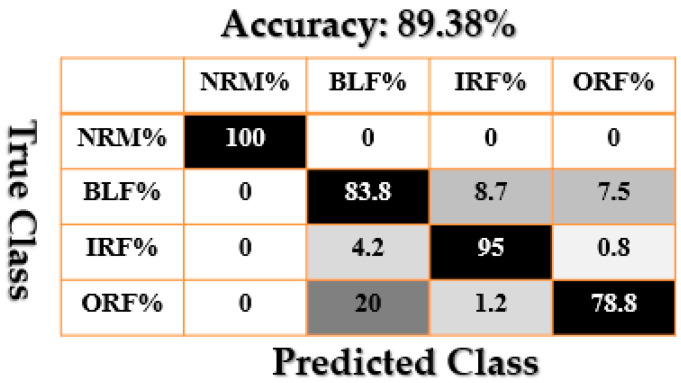
Fault pattern recognition confusion matrix based on the RAW + CNN.

**Figure 13:**
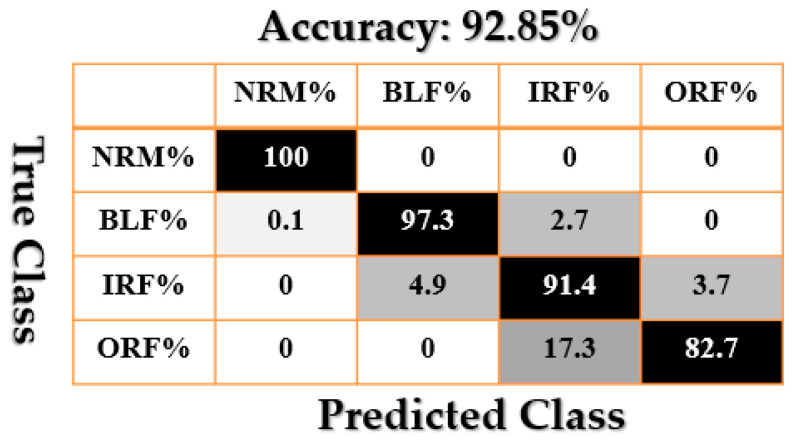
Fault pattern recognition confusion matrix based on the proposed + SVM.

**Figure 14:**
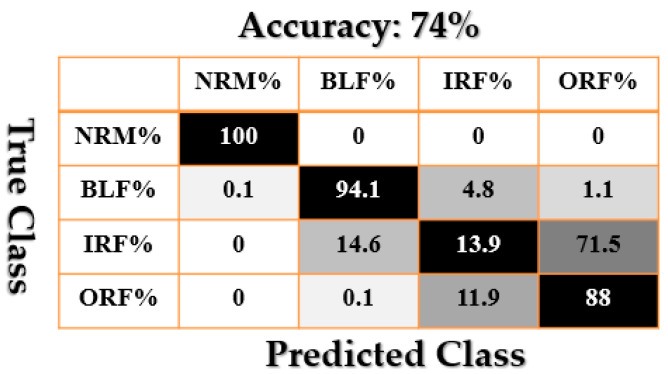
Fault pattern recognition confusion matrix based on the SVAL-HVSO + SVM.

**Figure 15:**
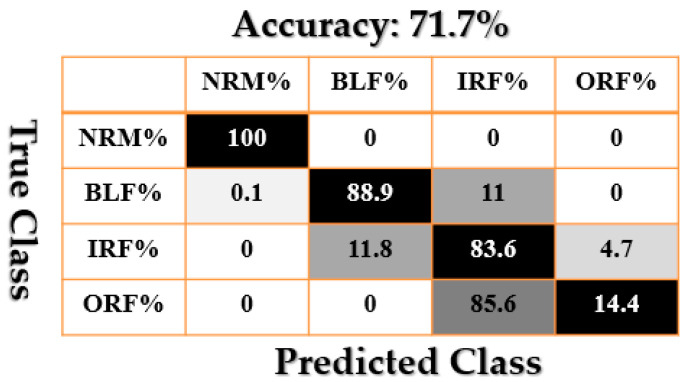
Fault pattern recognition confusion matrix based on the SVAL-VSO + SVM.

Figure 9, Figure 10, Figure 11, Figure 12, Figure 13, Figure 14 and Figure 15 show the confusion matrices of fault pattern recognition for bearing vibration signals based on the proposed + CNN, SVAL-HVSO + CNN, SVAL-VSO + CNN, RAW + CNN, SVAL-ANFS + SVM, SVAL-HVSO + SVM, and SVAL-VSO + SVM, respectively.

Figure 9, Figure 10, Figure 11 and Figure 12 illustrate the impact of the proposed estimation technique—the combination of the SVAL algorithm and adaptive neural-fuzzy structure + CNN for fault pattern recognition. To validate the power of CNN in these four methods, the SVM is used with the same training and testing data presented in Table 2. Figure 13, Figure 14 and Figure 15 illustrate the confusion matrices of fault pattern recognition for the SVAL-ANFS + SVM, SVAL-HVSO + SVM, and SVAL-VSO + SVM, respectively.

As regards Figure 9, Figure 10, Figure 11, Figure 12, Figure 13 and Figure 14, it is clear that the accuracy of fault pattern recognition for bearing vibration signals based on the proposed + CNN is better than the others. In addition, the average fault pattern recognition accuracy for the proposed + CNN is 99.48%, whereas in SVAL-HVSO + CNN, SVAL-VSO + CNN, SVAL-ANFS + SVM, SVAL-HVSO + SVM, and SVAL-VSO + SVM, the average fault pattern recognition accuracies are 97.71%, 97.08%, 92.85%, 74%, and 71.07%, respectively.

Table 3, Table 4 and Table 5 illustrate the power of crack size identification for the ball fault, inner fault, and outer fault, respectively, using the proposed + CNN, SVAL-HVSO + CNN, SVAL-VSO + CNN, RAW + CNN, SVAL-ANFS + SVM, SVAL-HVSO + SVM, and SVAL-VSO + SVM.

Regarding Table 3, it is clear that the accuracy of ball crack size identification for bearing vibration signals based on the proposed + CNN is better than those of the others. In addition, the average ball crack size identification accuracy for the proposed + CNN is 99.17%, whereas in the SVAL-HVSO + CNN, SVAL-VSO + CNN, RAW + CNN, SVAL-ANFS + SVM, SVAL-HVSO + SVM, and SVAL-VSO + SVM it is 95%, 92.08%, 77.5%, 92.3%, 59.3%, and 57.3%, respectively. Thus, the proposed algorithm improved the average ball crack size identification accuracy by 4.17%, 7.09%, 21.67%, 6.87%, 39.87%, and 41.87% compared to the SVAL-HVSO + CNN, SVAL-VSO + CNN, RAW + CNN, SVAL-ANFS + SVM, SVAL-HVSO + SVM, and SVAL-VSO + SVM, respectively.

Table 4 illustrates the power of crack size identification for an inner fault using the proposed + CNN, SVAL-HVSO + CNN, SVAL-VSO + CNN, RAW + CNN, SVAL-ANFS + SVM, SVAL-HVSO + SVM, and SVAL-VSO + SVM. As regards Table 4, it is clear that the accuracy of inner crack size identification for bearing vibration signals based on the proposed + CNN and the SVAL-HVSO + CNN is better than that of the others. In addition, the average inner crack size identification accuracy for the proposed + CNN is 100%, whereas in the SVAL-HVSO + CNN, SVAL-VSO + CNN, RAW + CNN, SVAL-ANFS + SVM, SVAL-HVSO + SVM, and SVAL-VSO + SVM it is 100%, 99.17%, 92.5%, 95%, 69.7%, and 56.8%, respectively. Thus, the proposed algorithm improved the average inner crack size identification accuracy by 0.83%, 7.5%, 5%, 30.3%, and 43.2% compared to the SVAL-VSO + CNN, RAW + CNN, SVAL-ANFS + SVM, SVAL-HVSO + SVM, and SVAL-VSO + SVM, respectively.

Table 5 demonstrates the power of crack size identification for an outer fault using the using the proposed + CNN, SVAL-HVSO + CNN, SVAL-VSO + CNN, RAW + CNN, SVAL-ANFS + SVM, SVAL-HVSO + SVM, and SVAL-VSO + SVM. Regarding Table 5, the accuracy of outer crack size identification for bearing vibration signals based on the proposed + CNN is better than those of the others. In addition, the average outer crack size identification accuracy for the proposed + CNN is 100%, whereas in SVAL-HVSO + CNN, SVAL-VSO + CNN, RAW + CNN, SVAL-ANFS + SVM, SVAL-HVSO + SVM, and SVAL-VSO + SVM it is 97.5%, 92.08%, 76.25%, 96.53%, 62.7%, and 56.7%, respectively. Therefore, the proposed algorithm improved the average outer crack size identification accuracy by 2.5%, 7.92%, 23.7%, 3.47%, 37.3%, and 43.3% compared to the SVAL-HVSO + CNN, SVAL-VSO + CNN, RAW + CNN, SVAL-ANFS + SVM, SVAL-HVSO + SVM, and SVAL-VSO + SVM, respectively. In summary, the average accuracies of fault pattern recognition and crack size identification based on the proposed + CNN are 99.48% and 99.72%, respectively. Thus, the proposed hybrid framework is suitable for accurate fault diagnosis of the bearing in different crack sizes and torque loads in comparison with the other referenced algorithms. From the experimental results, it is obvious that the application of the adaptive neuro-fuzzy structure observer, which is modeled by the SVAL approach, improves the power of the fault pattern recognition and crack size identification using CNN in comparison with the other observation techniques. However, in noisy conditions, the proposed signal modeling approach has limitations to modeling. To address this issue, the noise cancellation technique integrated with the signal modeling approach is suggested. However, the proposed method is reliable and robust, complexity is the next limitation compared to linear-based observers or artificial intelligence-based observers.

## 5. Conclusions

The principal purpose of this work was to solve the challenge of fault pattern recognition and crack size identification in a bearing. A support vector autoregressive–Laguerre scheme, an adaptive neural-fuzzy structure technique, and a CNN were combined to address this issue. This approach consists of three main steps. First, the signal was modeled in normal operation using a combination of the autoregressive technique, Laguerre algorithm, and support vector regression vibration signal approximation techniques. After modeling the normal vibration signal, the signal was estimated using a combination of the modern control algorithm and artificial intelligence techniques. To estimate the signal, first, the robust variable structure technique was selected, and in later stages, this procedure was modified. Next, the problem of the chattering phenomenon was solved by combining the variable structure technique with high-order super twisting. Then, to increase the flexibility and accuracy, the high-order variable structure technique was combined with a neural-fuzzy inference approach. In the final step, the fault was recognized and the crack size was identified using the CNN. The proposed algorithm improved the average fault pattern recognition accuracy by 1.77%, 2.4%, 10.1%, 6.63%, 25.48%, and 27.78% compared to the combination of the SVAL algorithm and high-order variable structure technique + CNN, the combination of the SVAL algorithm and variable structure technique + CNN, the original RAW signal + CNN, the combination of the SVAL algorithm and adaptive neural-fuzzy structure + SVM, the combination of the SVAL algorithm and high-order variable structure technique + SVM, and the combination of the SVAL algorithm and variable structure technique + SVM, respectively. In addition, the proposed method modified the average accuracy of crack size identification by 2.2%, 5.28%, 17.64%, 5.11%, 35.8%, and 42.79% compared to the combination of the SVAL algorithm and high-order variable structure technique + CNN, the combination of the SVAL algorithm and variable structure technique + CNN, the original RAW signal + CNN, the combination of the SVAL algorithm and adaptive neural-fuzzy structure + SVM, the combination of the SVAL algorithm and high-order variable structure technique + SVM, and the combination of the SVAL algorithm and variable structure technique + SVM, respectively. In future work, we will focus on improving the robustness, reliability, accuracy, and flexibility of the proposed work for fault diagnosis, fault prediction, and fault-tolerant control in different applications including rotating machines, robot manipulators, and pipelines. The possible directions for the improvement are discovering the robust function approximation using the combination of the dynamical-based procedure and data-driven-based approach. Furthermore, we will improve the power of flexibility and robustness of the estimation algorithm using a combination of nonlinear architecture of the observation approach and nonlinear architecture of the deep learning method in parallel. Additionally, the problem of highly noisy and uncertain signals should be considered, and the proposed procedure should be validated using the vibration and acoustic emission datasets.

## Figures and Tables

**Table 1:** Information of CWRU dataset [46].

Dataset Group	Condition	Load (hp)	Crack Sizes (in)
i	NRM	0	0.007, 0.014, and 0.021
	BLF	0	
	IRF	0	
	ORF	0	
ii	NRM	1	0.007, 0.014, and 0.021
	BLF	1	
	IRF	1	
	ORF	1	
iii	NRM	2	0.007, 0.014, and 0.021
	BLF	2	
	IRF	2	
	ORF	2	
iv	NRM	3	0.007, 0.014, and 0.021
	BLF	3	
	IRF	3	
	ORF	3	

**Table 2:** Details of dataset for training and testing the 1D-CNN.

			No. of Training Samples	No. of Testing Samples
Fault Pattern Recognition		NRM	960	240
		BLF	960	240
		IRF	960	240
		ORF	960	240
Crack Size Identification	Outer	0.007 mm	320	80
		0.014 mm	320	80
		0.021 mm	320	80
	Inner	0.007 mm	320	80
		0.014 mm	320	80
		0.021 mm	320	80
	Ball	0.007 mm	320	80
		0.014 mm	320	80
		0.021 mm	320	80

**Table 3:** Ball fault crack size identification and average accuracy using proposed + CNN, SVAL-HVSO + CNN, SVAL-VSO + CNN, RAW + CNN, SVAL-ANFS + SVM, SVAL-HVSO + SVM, and SVAL-VSO + SVM.

Crack Sizes (inch)	0.007	0.014	0.021	Average
**Proposed + CNN%**	100	97.5	100	99.17
**SVAL-HVSO + CNN%**	97.5	88.8	98.8	95
**SVAL-VSO + CNN%**	96.3	82.5	95.5	92.08
**RAW + CNN%**	78.8	73.8	80	77.5
**SVAL-ANFS + SVM%**	100	82	95	92.3
**SVAL-HVSO + SVM%**	55	41.3	81.8	59.3
**SVAL-VSO + SVM%**	78.3	13.5	80.3	57.3

**Table 4:** Inner fault crack size identification and average accuracy using proposed + CNN, SVAL-HVSO + CNN, SVAL-VSO + CNN, RAW + CNN, SVAL-ANFS + SVM, SVAL-HVSO + SVM, and SVAL-VSO + SVM.

Crack Sizes (inch)	0.007	0.014	0.021	Average
**Proposed + CNN%**	100	100	100	100
**SVAL-HVSO + CNN%**	100	100	100	100
**SVAL-VSO + CNN%**	100	100	97.5	99.17
**RAW + CNN%**	88.8	88.8	100	92.5
**SVAL-ANFS + SVM%**	98.5	92	94.3	95
**SVAL-HVSO + SVM%**	35.3	87.3	86.5	69.7
**SVAL-VSO + SVM%**	35.3	50.5	84.5	56.8

**Table 5:** Outer fault crack size identification and average accuracy using proposed + CNN, SVAL-HVSO + CNN, SVAL-VSO + CNN, RAW + CNN, SVAL-ANFS + SVM, SVAL-HVSO + SVM, and SVAL-VSO + SVM.

Crack Sizes (inch)	0.007	0.014	0.021	Average
**Proposed + CNN%**	100	100	100	100
**SVAL-HVSO + CNN%**	96.3	100	96.3	97.5
**SVAL-VSO + CNN%**	85	97.5	93.8	92.08
**RAW + CNN%**	51.3	98.8	78.8	76.25
**SVAL-ANFS +SVM%**	99.3	95	95.3	96.53
**SVAL-HVSO + SVM%**	36.8	74	77.5	62.7
**SVAL-VSO + SVM%**	36	73.5	60.5	56.7

## Data Availability

The data are publicly available.

## Abbreviations

CNN	convolution neural network	CWRU	Case Western Reverse University
SVM	support vector machine	AR	autoregressive
ARX	autoregressive with external input	PI	Proportional Integral
SVR	support vector regression	SVAL	support vector autoregressive–Laguerre
HOVSO	high-order variable structure observer	ANFIS	adaptive neural-fuzzy inference system
ANFSO	adaptive neural-fuzzy structure observer	NRM	normal condition
BLF	ball fault	IRF	inner race fault
ORF	outer race fault	AL	autoregressive Laguerre
UPI	uncertainty performance index	Proposed + CNN	combination of the SVAL algorithm and adaptive neural-fuzzy structure (proposed) + CNN
SVAL-VSO + CNN	combination of the SVAL algorithm and variable structure technique + CNN	SVAL-HVSO + CNN	combination of the SVAL algorithm and high-order variable structure technique + CNN
Proposed + SVM	combination of the SVAL algorithm and adaptive neural-fuzzy structure (proposed) + SVM	SVAL-VSO + SVM	combination of the SVAL algorithm and variable structure technique + SVM
RAW + CNN	Combination of the RAW signal + CNN	SVAL-HVSO + CNN	combination of the SVAL algorithm and high-order variable structure technique + CNN
